# Understanding the Relationship between Intention and Cat Containment Behaviour: A Case Study of Kitten and Cat Adopters from RSPCA Queensland

**DOI:** 10.3390/ani10071214

**Published:** 2020-07-16

**Authors:** Lynette J. McLeod, Di Evans, Bidda Jones, Mandy Paterson, Sarah Zito

**Affiliations:** 1 School of Psychology, University of New England, Armidale, NSW 2350, Australia; 2 RSPCA Australia, PO Box 265, Deakin West, ACT 2600, Australia; DEvans@rspca.org.au (D.E.); bjones@rspca.org.au (B.J.); SZito@rspca.org.au (S.Z.); 3 Sydney School of Veterinary Science, University of Sydney, Sydney, NSW 2006, Australia; 4 RSPCA Queensland, Locked Bag 3000, Archerfield BH, Qld 4108, Australia; mpaterson@rspcaqld.org.au

**Keywords:** human behaviour change, domestic cat management, intervention development

## Abstract

**Simple Summary:**

People shape an intention to perform a certain behaviour from a positive assessment of that behaviour, which in turn creates a level of desire, and impulse, to do it. Whether or not this behaviour is actually performed depends on competing plans, evaluations, motives and impulses. This study explored the relationship between the intentions and actual cat containment behaviours of 72 cat adopters from a RSPCA Queensland animal shelter. We found that the cat containment intentions of these participants only moderately predicted their containment behaviour, and identified a number of important factors that prevented some of the participants from containing their cat once they got it home. The results from this research will be used to guide the development of additional targeted strategies to assist individual cat owners contain their pet.

**Abstract:**

In Australia, cat owners are encouraged to keep their pet cats contained on their property at all times. This study explores the relationship between the intentions and behaviours of 72 kitten and cat adopters from a RSPCA Queensland animal shelter, to provide a more in-depth understanding of the factors influencing the adoption of cat containment behaviours. At the time of adoption, 64 participants (89%) indicated they were intending to keep their cat fully contained. Eight weeks after adoption, 63 participants (87%) reported they were doing so (59 who had stated their intention at the time of adoption, and 4 who had not). We found cat owner containment behaviour was moderately correlated with containment intentions. For some of the participants when it came to enacting this behaviour, their intentions and the provided education information was not enough to overcome the more compelling capability, opportunity and motivational factors which presented themselves once they got home. We were able to identify these factors and suggest additional behaviour change strategies that would assist. Although it is important to provide cat adopters with advice about how to contain their cats properly, these results also highlight the importance of focusing attention on other behaviour change strategies that address the particular barriers faced by some cat-owners who are unsuccessful in keeping their cat contained on their property.

## 1. Introduction

Various campaigns encouraging the containment of pet domestic cats, *Felis catus*, have been operating in Australia since the early 1990s. Advocates for these campaigns give a range of reasons for cat containment including: (1) Health and welfare benefits for the cats, such as the reduction in the risk of serious injury from traffic, fighting, dogs and acts of cruelty by humans, the reduced spread of cat-specific diseases and the prevention of unwanted pregnancies (e.g., [1,2]); (2) community benefits with the reduction of nuisance disturbances and neighbour disputes [3]; (3) conservation benefits, with the potential predatory nature of any free-roaming cat, regardless of their ownership status, being implicated in the decline of local wildlife populations [4]; and (4) public health benefits with the reduction in the transmission of diseases, such as *Toxoplasma gondii*, and faecal pollution of waterways [5,6].

In 2019, the number of pet cats in Australia was estimated to be almost 3.8 million (no change from 2016) [7,8]. Around a third of these cats (34%) are kept exclusively indoors, with the majority (59%) kept both indoors and outdoors, and 7% kept exclusively outdoors (note: it is not specified in this report whether, when outdoors, the movements of these cats are restricted or not). A quarter of these cats are reported to have been adopted from animal shelters, mainly run by cat advocacy groups and animal welfare organisations such as the RSPCA and Animal Welfare League [7].

RSPCA Australia recommends that cats should be contained to the owner’s property. This can be indoors, preferably with access to an outdoor enclosure. Promotion of cat containment by the RSPCA focuses on owners ensuring that their cat’s physical and mental needs are met as, without achieving this, the welfare of a contained cat may be compromised [9]. A recent report of best-practice domestic cat management in Australia released by the RSPCA recommended that “Education programs are needed to increase the acceptance and uptake of “24-h” cat containment, with subsequent regulation in areas of high conservation value” [3].

### 1.1. Changing Human Behaviour

Awareness and knowledge, through educational information, are arguably the first requirements for changing human behaviour [10]. If individuals do not know that there may be an issue with their current behaviour and why this may be the case, how are they expected to contemplate a change? Intentions to then perform a certain behaviour are generated from a positive evaluation of this behaviour, which in turn creates a level of desire, and impulse, to do it. Whether or not this behaviour is actually performed depends on competing plans, evaluations, motives and impulses or inhibitions at the time (PRIME theory [11]). Thus, a cat owner may have the good intentions to contain their cat, but when it comes to enacting this behaviour, they may face a range of competing internal and external factors which ultimately prevent this plan coming to fruition.

A large number of behaviour models have been developed to help us understand the underlying factors influencing an individual’s behaviours. For example, Michie and her colleagues describe 83 behaviour theories relevant for health issues alone [12]. This suggests that there can be a very large number of potential factors that can either encourage the behaviour (drivers) or impede it (barriers). McLeod and colleagues [13] described that most drivers and barriers relevant to understanding cat management can be classified into the three categories described by Michie and her colleagues in their overarching integrative model of behaviour, the Capability, Opportunity, Motivation-Behaviour (COM-B) model [14,15]. According to the model, behaviour is determined by three main factors:

1. *Capability*. An individual’s physical and psychological capacity to engage in the behaviour of interest. COM-B distinguishes between two types of capability. *Physical capability* refers to the extent to which an individual can engage in the behaviour. For example, does an individual have the physical ability to install a cat-proof fence? *Psychological capability* refers to the capacity to engage in the necessary mental activities (risk assessments, mental simulation of possible outcomes, decision making, etc.) to select and implement an appropriate course of action.
2. *Opportunity*. These are factors external to the individual that prompt or enable the behaviour to occur. COM-B distinguishes between two types of opportunity. *Physical opportunity* refers to situational factors such as having relevant equipment or supplies readily available that are needed to address the problem. *Social opportunity* refers to cultural or community values and norms that may make engaging in recommended best practices more or less likely. For example, if most cat owners within a neighbourhood are keeping their cats in at night, this creates a social norm that increases that likelihood that others in the neighbourhood will also engage in this practice.
3. *Motivation*. These are factors internal to the individual that energise or direct behaviour. COM-B distinguishes between two types of motivational factors [16]. *Reflective motivation* consists of conscious deliberation and reasoning, and often involves evaluating threats, planning, goal setting, and mentally simulating possible outcomes associated with various types of actions. For example, prior to deciding how to contain their cat, an owner may make a list of the costs and benefits of engaging and not engaging in the practice, and select the option that he or she believes is most likely to produce the most positive outcome. *Automatic motivation* refers to mental processes that operate largely outside conscious control of the individual, including habits, impulses and emotionally driven behaviour. For example, a cat owner’s “decision” to keep their cat contained may be emotionally based by witnessing the injuries suffered by their cat after being hit by a car.

Thus an individual’s capabilities, current physical and social opportunities, and motivations can have a firmer hold on an individual’s view on life as they contemplate whether they need to change their behaviour, then initiate and maintain the appropriate action [11,15,17,18].

One of the advantages of using this COM-B model is that it allows practitioners to link the identified COM-B mechanisms that drive or impede the desired behaviour, i.e., capability, opportunity or motivation, to the most appropriate behaviour change techniques. For example the best techniques to use when capability factors are identified include education, training, or helping. When opportunity is identified an intervention will need to provide, enable, facilitate, offer, prompt or constrain. Motivation factors are best tackled by informing, discussing, persuading, demonstrating incentivising or coercing [11,14,15,17,18,19].

### 1.2. Aims of This Study

This study investigated the cat containment behaviours of new kitten and cat adopters at a RSPCA Queensland animal shelter. It had three main objectives:

1. Measure the intention of new adopters to contain their newly adopted cat and assess whether they followed through with this intention,
2. Compare the response to the information given at the time of adoption provided as a printed booklet and/or as an online link,
3. Further understand the behavioural factors (capabilities, opportunities and motivations) that influence those adopters who do not contain their cats.

We take an idiographic approach, exploring the actions of specific individuals to provide a more in-depth understanding of the factors influencing cat containment behaviour of cat adopters, and the efficacy of an education intervention [20,21]. The results from this research will be used to better understand RSPCA cat adopters, and help identify barriers to cat containment which can be used to guide the development of more targeted strategies to assist individual cat owners contain their pet.

## 2. Materials and Methods

One hundred and seventeen participants were initially recruited through the RSPCA Queensland Animal Care Campus at Wacol between September 2018 and April 2019. Having made the decision to adopt a kitten or cat, cat adopters were invited to participate in the research study, and provided with an information sheet to read and a consent form to sign as per the Human Ethics requirements (all procedures performed in studies involving human participants were in accordance with the ethical standards of the Human Research Ethics Committee of the University of New England-Approval No. HE18-043). Before receiving any information on cat containment, participants’ intention to contain their newly adopted cat was documented.

Two groups were created by allocating adopters randomly by day of adoption, i.e., every participant on a particular day was allocated to the pre-determined, randomly generated, group for that day. The adoption counsellors were informed at the start of the day as to which group had been allocated for that particular that day, i.e., treatment or control.

The control group (C) received the current standard adoption information (N = 51). This involved the adoption counsellor outlining the processes for introducing animals to a new home, as well as the new owner’s obligations, including the RSPCA recommendation that cats be always contained to their owner’s property, either indoors and/or outdoors in a secure cat enclosure or cat escape-proof yard. The participants were not given any printed information, but were emailed a link to the RSPCA Queensland’s website (https://www.rspcaqld.org.au/what-we-do/provide-animal-care-advice) where they could access a range of fact sheets containing further information about many issues, including cat containment.

In addition to the standard adoption information as described above for the control group, each participant in the treatment group (T) (N = 66) was given a personal printed copy of the RSPCA 22-page booklet “Keeping your cat safe and happy at home” (S1). The information contained in this booklet was more detailed and comprehensive than that provided on the website, and covered the reasons why they should contain their cat, the options available, and how to create an appropriate environment for their cat. The content design for this booklet was guided by previously identified drivers and barrier from the literature [22,23,24,25,26].

All adoption counsellors, whether staff members or volunteers, were trained before the commencement of the study, and instructed to take the same time with the adoption interview, irrespective of treatment group. The standard adoption process includes a discussion to assess the suitability of a particular cat for a particular home. The interview generally took approximately 10 min, however some cats may have necessitated an extra veterinary consult as well. All cats and kittens offered for adoption by the RSPCA were neutered.

Eight weeks after adoption, participants were sent an email with an invitation to complete an online survey. Eight weeks was chosen as a suitable time to allow new owners to act on motivations that they might already have had or were triggered by the information provided to them at the time of adoption, but not too long that they had forgotten the material and would therefore be unable to assess its usefulness. This survey collected information on the participants’ demographics (age, gender, locality, home ownership, dwelling type, access to outdoor space, household membership, and other types of pets) and current cat containment behaviour. Participants were asked to rate, using a 5-point Likert scale (1 = do not agree, 2 = slightly agree, 3 = somewhat agree, 4 = agree, 5 = strongly agree), seven potential drivers (motivations) for cat containment, and 14 barriers (mixture of capability, opportunity and motivation) to cat containment that had been identified from the literature [22,23,24,25,26]. These behavioural factors are listed in Table 1. Those participants who indicated they were not fully containing their cat at 8 weeks post-adoption were asked about their future intentions. There was opportunity for the participants to provide further details in three open-ended questions: The “benefits of keeping their cat on their property all the time”, and whether they had “experienced any problems associated with containing their cat”, or “the reasons why they are not keeping their cat contained to their property all the time”.

Participants were also asked whether they had read any of the information that was provided, and those in the treatment group were asked to rate the booklet they had received, using four measures: (1) How easy the information was to understand, (2) how credible they found the information, (3) how clear the steps were to follow, and (4) how encouraging the information was to contain their cat. A 5-point Likert scale (1 = do not agree, 2 = slightly agree, 3 = somewhat agree, 4 = agree, 5 = strongly agree) was used. All questions had been piloted to reduce any ambiguity and ensure subjects understood the terminology. A copy of the survey is available in Supplementary Materials (S2).

If after two weeks the participants had not completed the survey, they were sent a second email to prompt their participation, followed by a phone call a week later (if they supplied their phone details and had consented to be contacted this way). As an added incentive to complete this survey, a chance to win a cat “pamper pack” valued at AU$200 was offered, with all respondents being eligible.

Two of the initial recruited participants had returned their adopted cat to the RSPCA within the eight-week period. Seventy-four adopters (47T, 27C) responded to the invitation to complete the online survey (63% response rate).

### Statistical Analysis

Bivariate correlations were calculated to examine inter-relationships between the containment, demographic, driver and barrier variables. One-way ANOVA and Pearson’s chi-squared test was used to test for differences between intentions of participants who responded to the online survey, and the other recruited participants who did not, differences between intentions and behaviour after 8 weeks, as well as differences between containment profiles. All statistics were calculated in SPSS v25 [27].

## 3. Results

### 3.1. Participants

The 74 participants who completed the online survey consisted of 56 females and 18 males, with an average age of 36.5 years. More than half (43) owned their own home, while the remaining 31 rented. Nearly a third (23) already owned another cat (average 1.1, range 1–2), while 28 owned other types of pets (mainly dogs). Thirty-two of the households consisted of adults and children, with 41 households having only adults present, and one declining to answer this question. Nineteen lived in the inner city, 52 in the suburbs and 3 in semi-rural areas. Forty-five lived in stand-alone houses, 10 in duplexes/townhouses, and 19 in an apartment/flat. Three had no access to outside space, 14 had balconies, 14 had courtyards, 40 had backyards and 3 had acreages. Our sample is similar to that of Australian cat owners as reported by Animal Medicines Australia in their Australia-wide survey of pet owners from both urban and regional areas [7]. They reported that 79% of cat-owners are female (our study sample 76%), 65% are homeowners (our study sample 58%), 30% live in households with children (our study sample 43%), and 68% live in free-standing homes (our study sample 61%).

We checked for any differences in the containment intentions between the 74 participants who responded to the online survey after 8 weeks, and the other 43 cat adopters who were initially recruited but did not complete the online survey to rule out any bias. We found no significant difference (X^2^ = 5.44, df = 6, p = 0.49). On the day of adoption, 102 of the recruited participants (87%: 56T, 46C) stated they were intending to keep their newly adopted cat fully contained, six (5%: 4T, 2C) were intending to keep their cat contained only at night, and nine (8%: 6T, 3C) had no plans to contain their cat. Of the 74 participants who completed the online survey, 66 (89%: 42T, 24C) had stated they were intending to keep their newly adopted cat contained to their property, four (5%: 2T, 2C) were intending to keeping their cat contained only at night, and four (5%: 3T, 1C) had no plans to contain their cat.

### 3.2. Containment Intentions and Behaviour

Three cat containment profiles were identified after 8 weeks: (1) Fully contained (N = 63 (85%): 38T, 25C), where the cat was either kept indoors all the time (not allowed outside), or indoors with restricted outdoor access (enclosure, cat escape-proof yard, or on a leash), (2) Contained at night only (night curfew) (N = 5 (7%): 4T, 1C), where the cat was kept indoors at night and allowed to roam unrestricted during daylight hours, and (3) Not contained (N = 4 (5%): 3T, 1C) where the cat was allowed to roam unrestricted all the time. Two (2T) of the participants declined to answer the questions related to current containment behaviour. For those 63 owners who were fully containing their cats, 39 (62%) were keeping their cat indoors all the time (not allowed outdoors), and the remaining 24 were keeping their cat indoors with restricted outdoor access (enclosure, cat escape-proof yard, or on a leash).

**Figure 1:**
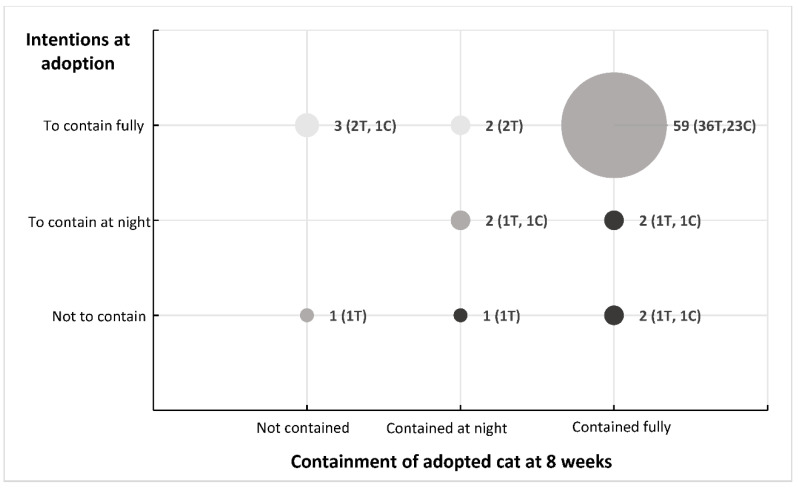
Summary of the containment intentions of the 72 participants at the time of adoption (vertical axis), and their containment behaviour after 8 weeks (horizontal axis). Size of dots represent number of participants. The lighter dots represent participants who did not follow through on their intentions, the darker dots represent those participants who surpassed their initial intentions. T = treatment, C = control.

Participants’ containment intentions at the time of adoption were moderately correlated with their cat containment profile at eight weeks (r = 0.32) [28]. A summary of the differences between containment intentions at adoption and containment behaviour at 8 weeks of these 72 participants is shown in Figure 1. Ninety two percent of participants who expressed their intention to keep their cat contained at point of adoption were doing so after 8 weeks. For the 4 participants who had not intended to contain their cat, 3 of them were actually performing some level of containment.

The nine participants who indicated they were not fully containing their cat after 8 weeks were asked about their future intentions to do so. Responses ranged from ‘definitely not’ (N = 6; 5T, 1C), “probably not” (N = 2; 1T, 1C) to maybe (N = 1; 1T). No positive responses (“probably yes” or “definitely yes”) were recorded.

### 3.3. Influence of Previous Behaviour on Containment

Twenty two of the *72* participants who had completed the containment questions indicated they owned other cats. Two participants indicated they were not containing their other cats, two were keeping these other cats in at night only, and the remaining 18 were fully containing their other cats. The remaining 50 participants did not have another cat currently residing in their household. Both intention to contain the newly adopted cat and containment behaviour at 8 weeks were strongly correlated with containment of the other owned cats (intention: r = 0.56; at 8 weeks: r = 0.89) [28].

**Figure 2:**
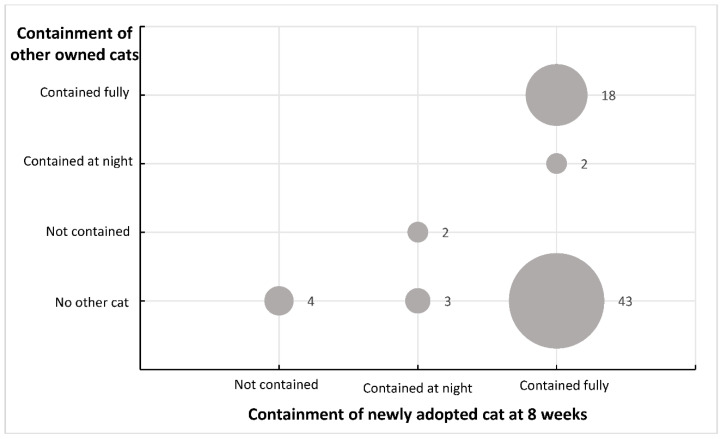
Summary of the containment behaviour for other owned cats (vertical axis), and the containment behaviour for the newly adopted cat after 8 weeks (horizontal axis). Size of dots represent number of participants.

A summary of the differences between containment of other owned cats and containment of the newly adopted cat at 8 weeks is shown in Figure 2. All four participants who reported they were not containing their newly adopted cat did not have another cat residing in their household. Two participants who reported they were keeping their newly adopted cat contained only at night had cats that they were currently not containing. Two participants who were fully containing their newly adopted cat, owned another cat which they only contained at night.

### 3.4. Influence of Provided Information on Containment

Three participants (2T, 1C) did not respond to the questions about the online information or the booklet that they received at the time of adoption. Over half of the participants (24T, 14C) made at least some use of the online information (accessing and reading some or all of the information). The remaining participants either did not recall receiving the email (11T, 9C) or did not access the information, i.e., click on the link or read the content (10T, 3C).

Four of the five participants who expressed their intention to fully contain their cats but at 8 weeks were either not containing, or containing at night only (represented by the light dots in Figure 1) reported that they had accessed and read some or all of the containment information on the website, and the booklet (for those in the treatment group). The remaining participant did not recall receiving the email; however, they did read some of the information contained in the booklet.

The three participants who had not expressed an intention to contain their cat but at 8 weeks were containing, either fully or at night (represented by the bottom row of dark dots in Figure 1), had accessed and read some or all of the containment information on the website. Those in the treatment group had read some or all of the booklet. Both the participants who had expressed an intention to contain their cats at night but at 8 weeks were fully containing their cats (represented by the dark dot in the middle row in Figure 1), did not access the information on the website. The one in the treatment group did not recall receiving the booklet.

Only participants in the treatment group who had read some, or all of the information in the provided printed booklet (N = 31) were asked to rate the booklet’s content. Surprisingly, those participants who were not containing their cat rated all four measures (ease of understanding, credibility, how clear the steps were to follow and how encouraging the information was to contain their cat) fairly highly, along with those participants who were fully containing their cats (Table 2). Participants who were only containing their cats at night rated each measure slightly lower. These differences could not be statistically compared owing to the small group sizes.

The four participants in the treatment group who expressed their intention to fully contain their cats but at 8 weeks were not containing, or containing only at night (represented by the light dots on Figure 1) rated all four measures highly (rating scores 4 and 5′s). All four measures were also rated highly by the two participants in the treatment group who had not expressed an intention to fully contain their cats but at 8 weeks were either fully containing or containing their cat at night (represented by the bottom row of dark dots on Figure 1). One participant in the treatment group who had expressed an intention to contain their cat at night but at 8 weeks were fully containing their cat (represented by the dark dot on the middle row in Figure 1) did not recall receiving the booklet, so did not rate its content.

There were high intentions to contain cats and containment adoption across both treatment and control groups. Therefore, we were unable to detect any influence on the change of intention or uptake of containment of providing the printed booklet as opposed to only supplying a link to online information (change: *X*^2^ = 1.25, df = 3, *p* = 0.74; uptake: *X*^2^ = 1.02, df = 1, *p* = 0.31).

### 3.5. Other Influential Factors on Containment Behaviour

**Figure 3:**
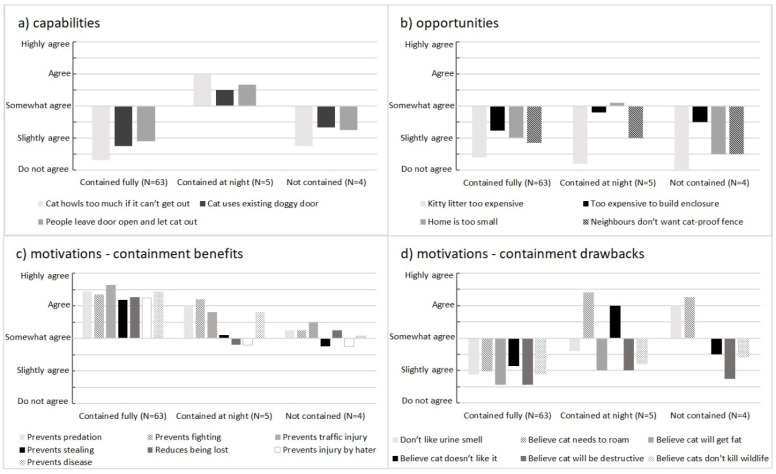
Average agreement scores (5 = highly agree, 4 = agree, 3 = somewhat agree, 2 = slightly agree, 1 = do not agree) to the 20 identified driver and barrier factors (**a**) 3 capabilities, (**b**) 4 opportunities, (**c**) 7 positive motivations, and (**d**) 6 negative motivations) for participants keeping their cat fully contained (N = 63), contained at night only (N = 5) and not contained (N = 4). Refer to Table 1 for the list and description of these factors.

Participants’ agreement with the 20 identified influential factors (drivers and barriers) across the three containment profiles (fully contained, contained at night, not contained) are shown in Figure 3, and their demographic details are given in Table 3. There were notable differences in the capabilities, opportunities and motivations between these three containment profiles. There were no differences found for any of the demographic variables (gender, household, home ownership, building type, access to outdoor space) except for the ownership of other pets. Participants who owned pets other than dogs (e.g., birds and reptiles) were less likely to fully contain their cat, and more likely not to contain their cat at all (Table 3).

Participants who were fully containing their cats were more likely to agree with the benefits of containing their cats (as represented by positive values in Figure 3), and were less likely to agree with any of the identified barriers factors (as indicated by the values below the line in Figure 3). The participants who had either not expressed an intention to contain their cats, or to only contain their cats at night but at 8 weeks were fully containing (represented by the right column of dark dots on Figure 1) reported on the problems they had overcome to keep their cat contained:

*“[The cat] likes to scratch furniture, he gets feisty and wants to hunt/play/exercise. Excessive meowing. When feisty he is prone to love biting and scratching us”.*

*“Occasionally he will scoot out the front door when letting the dog out (also an indoor dog), but we’re quick to retrieve him or supervise short exploration in the yard. He’s still a kitten so very curious”.*

*“It’s [containment’s] against cat’s nature”.*

The key factors influencing the four participants who were containing their cats at night after 8 weeks (as represented by positive values in Figure 3) are:

- Reduced perceived capability as the cat howls too much if unable to get out, the cat uses existing doggy-door to get out and people leave doors open,
- Do not have the opportunity as they consider their home is too small to keep their cat inside, and
- strongly motivated by their beliefs that cats need to roam, and cats do not like being contained, as well as their beliefs about the benefits of containment (such as prevent predation, fighting with other cats, traffic injuries and spread of disease).

The participant who had not expressed an intention to contain their cat, but at 8 weeks was containing their cat at night (represented by the dark dot on the middle column on Figure 1) commented:

*“I think female cats roam less and I feel they need to roam around in nature for their own interest”.*

The participants who had expressed their intention to contain their cat at night only, and were doing so at 8 weeks commented:

*“We tried keeping him in at the beginning but every time we would open a door to the outside world he would run through it and if we stopped him and kept him inside he constantly meowed in a way that felt like he was screaming at us”.*

*“We lock our cat up at night. We let her out throughout the day as our home is small and gets hot through summer”.*

The participants who had expressed their intention to fully contain their cats but at 8 weeks were containing their cats only at night (represented by the light dot on the middle column on Figure 1) commented:

*“We like to give them a bit freedom to explore, they always came back at night for food and sleep”.*

*“He is out in the day and inside at night. He is out there living his best life”.*

Participants who were not containing their cats were highly motivated by their beliefs that cats need to roam and their dislike of the smell of urine in the house (as represented by positive values in Figure 3). This was despite their beliefs about the benefits of containment (such as prevent predation, fighting with other cats, traffic injuries and becoming lost).

One of the participants who had expressed their intentions to contain their cats but at 8 weeks was not containing their cat (represented by the dark dot in the left column in Figure 1) commented:

*“Believe that animals need to be outside for their general mental health and wellbeing (this includes humans!), also do not like kitty litter within house as it smells and harbours disease”.*

The other participant mentioned an additional barrier that we had not identified:

*“[Have an] old house with no screens on doors or all windows—so hard to contain [cat] in the heat”.*

Instead of containment they had placed a cat bib and bell on their cat believing this would reduce the impact on wildlife:

*“We have bought a cat bib and the noisiest bell we could find from the RSPCA shop. Our cat brings in lizards (alive) which we release, but so far no birds…”*

The participant who had not expressed an intention to contain their cat, and at 8 weeks was not containing their cat commented:

*“She’s happy, gives her fresh air and explore our backyard”.*

## 4. Discussion

This study explored the cat containment behaviour of adopters of new kittens and cats from a RSPCA Queensland animal shelter. In particular, we: (1) Measured the intention of the participants to contain their newly adopted cat and assessed whether they followed through with this intention, (2) compared the responses to information provided as a printed booklet and/or as an online link, and (3) further explored the factors that influence those adopters who did not fully contain their cats. We found the containment of the newly adopted cat was moderately correlated with containment intentions expressed at the time of adoption. For those participants who already owned a cat, containment of the newly adopted cat was highly correlated with the containment behaviour already practiced. Eighty-nine percent of the participants expressed their intention to keep their cat fully contained at the time of adoption. When it came to enacting this behaviour, only five of these participants (7%) were unable to overcome more compelling factors (capabilities, opportunities, or motivations), which prevented them following through with their initial intention. Four participants who initially did not express an intention to fully contain their cat, were doing so, with a further participant adopting a night curfew for their cat.

The actions of the nine participants who were allowing their cats to roam unrestricted as well as those keeping their cats in only at night were both strongly motivated by their beliefs that cats needed to roam. The four participants not restricting their cats at all were further motivated by their dislike of the smell of cat urine in the house. Instead, those adopting a night curfew for their cat appeared to be balancing their beliefs about the positive benefits of containment (preventing predation, fighting, injuries and disease transmission) with their belief that their cat did not like being contained and their inability to implement an effective containment strategy. The main capability barriers that were cited by all participants included preventing the cat escaping through doors, getting visitors to shut the doors, and providing suitable enrichment.

Cat curfews have been a popular management solution for local authorities, as they are easier to implement than full containment and appear to have a greater acceptance by the community [13]. However, their overall impact in reducing the negative impacts of cats is disputed [29]. Injuries to cats and disease transmission remains possible during daylight hours. In addition, wildlife predation is not necessarily reduced, only the wildlife species impacted varies [30,31,32,33]. The results from this study potentially support the strategy that getting some people, in the short term, to initially adopt a cat curfew may be the first step to adopting full containment in the longer term [13]. Although containment behaviour of their new cat could be motivated by a range of reasons, two of the participants in this study who were not fully containing their previously owned cat, opted to keep their newly adopted cat in at night. Two other participants who were keeping their previously owned cat contained at night, opted to fully contain their newly adopted cat.

One participant who was allowing their cat to roam unrestricted was using a bell and a cat bib on their cat in the hope of reducing their impact on wildlife. This is another contentious issue, with studies both supporting and rejecting the effectiveness of these methods [29,34,35,36,37,38]. In the push for the containment of pet cats, the use of curfews, and bells/bibs are two issues that need to be clarified (and communicated)—are they acceptable forms of management to help minimise the impacts on wildlife; what is their impact on the welfare of the cat; and what effect, if any, do they have on the other motivations for containing cats?

The proportion of participants keeping their newly adopted kitten or cat fully contained in this study was higher than has been previously reported, with 87% reporting that they were fully containing their cat. Reports published in the first half of this decade showed around a third of cat owners were keeping their cats fully contained [25,26]. This was an increase on the figures published in the 2000s, which showed approximately a quarter of cat owners were keeping their cat fully contained [39]. Thus, the relatively high number of owners containing their cat in this study may be a continuation of this shift in public opinion towards supporting containment due to the continuing publicity and awareness campaigns around cat containment. The high intentions to fully contain cats recorded at the time of adoption may also be an indication that the participants were aware of the need to contain their cat and had already read some information about cat containment from other sources. They might also be aware that the RSPCA recommends cat containment and this knowledge could influence how they answered questions. Another factor influencing the high containment rates reported in this study may be that the sample population was not representative of the general population of cat owners. Zito and her colleagues [40] found that people who seek to adopt from an RSPCA cat shelter are more likely to have put considerable thought into the adoption, and may also be keen to be seen to “do the right thing” as responsible owners.

Given the higher than expected rate of full cat containment, it was not possible to assess and compare the impact of the different educational materials as initially intended. Knowledge alone does not change behaviour, it also depends on the influence and impact that this new knowledge brings. The literature has many examples where interventions that provide only general education content often fail to produce significant behaviour change [41,42,43]. Cognitive biases, such as confirmation bias where we tend to favour and recall information that confirms or support our prior personal beliefs or values, will also affect the impact of educational material on behaviour change [16,44,45]. Providing educational information does not necessarily mean individuals will read it. We found that not everybody in the treatment group read the provided printed booklet, nor had everybody in either the treatment or control groups accessed and read the website information. More participants read some or all of the printed booklet than read the website information, but the difference was not significant. This suggests that more than just providing information is needed to overcome the barriers that are preventing them from adopting the full containment behaviour.

An important element of designing effective behavioural interventions is to match identified barriers to specific behaviour change techniques [15,17,46]. Behaviour change techniques best suited to changing the types of barriers identified by those participants not keeping their cat fully contained include: Training, personal advice and support, prompts, subsidies or provision of low cost options for cat containment structures, and feedback and/or demonstration from peers about their containment issues and solutions [11,14,15,17,18,19]. A novel promotion by the RSPCA may be to develop a sign/sticker for households to alert visitors about their contained cat. This would prompt them to take care when entering and to shut the door so the cat cannot escape. Alternatively, the owner could use a distraction technique to occupy the cat when using a door (e.g., train them to stay somewhere away from the door when asked or when the door opens, using reward-based training). Other ideas include crafting messages using persuasive communications techniques such as engaging message framing, story-telling and social norms [47], creating videos (e.g., https://www.kingborough.tas.gov.au/2018/03/inside-with-cats) to demonstrate how some cat owners are successfully containing their cat, creating and managing blogs (e.g., http://www.safecat.org.au/blog.html) to offer advice and support for people having problems with particular issues such as how to deal with howling cats, how to train their cat to walk on a leash, or creating more portable, affordable solutions to outside containment that can be used by people renting accommodation.

### Limitations and Future Research

As it took an idiographic approach, the results from this study are not able to be generalised. This is one of the few studies that has attempted to evaluate the effectiveness of an intervention aimed at changing people’s behaviour regarding containment of their cat. Although a rigorous experimental design was attempted, the reality of working with “real” people in the “real” world did create some limitations. It is difficult to truly randomise the treatments, and as adopters had to initially consent to be involved in the project, there is the potential for bias in the initial recruitment phase. Using an online survey to collect data may be another source of bias [48]. In our case, the containment intentions of our final sample of 72 participants did not differ significantly from those of the other recruited participants that did not complete the survey.

The reliability and quality of self-reports has been questioned as it is thought that respondents may tend to report what they believe the researcher expects, or report what reflects positively on themselves [49]. However, Chan [50] argues that although some respondents may be driven by social desirability, and provide the researchers with inaccurate data on some occasions, it does not happen all the time, and this issue is less serious in measures used in field studies and naturalistic settings. To counter these potential issues, all responses to our online survey were anonymous, and we framed our research as “improving the information provided to RSPCA cat adopters” and not on the need for cat containment per se, thus minimising the expectations of adopters towards this behaviour. Furthermore, we used well-established measures of psychological constructs where possible. We acknowledge that there may have been bias due to the participant’s ability to recall the provided information [51], as it was not feasible in this study to ask this question at a time soon after the participants had read and/or made use of this information. We also acknowledge that there may be a possibility that the general nature of the containment barrier and benefits questions that were asked may have influenced the participant’s response. Future research needs to address this question.

This study has highlighted the need for further research into the effectiveness of the suggested interventions to improve adoption of cat containment. Future research is also required into enrichment options offered to indoor cats and to determine if these cats appear to be happy and content by their owners.

## 5. Conclusions

This study used an idiographic approach to explore the relationship between the intentions and actions of specific individuals to provide a more in-depth understanding of the factors influencing cat containment behaviours of kitten and cat adopters from a RSPCA Queensland animal shelter. At the time of adoption, 89% of participants indicated they were intending to keep their cat fully contained. Eight weeks after adoption, 87% of participants reported they were doing so. We found the containment of the newly adopted cat was moderately correlated with containment intentions expressed at the time of adoption. For some of the participants, when it came to enacting this behaviour, intentions were not strong enough to overcome the more compelling capability, opportunity and motivational factors which presented themselves once they got home. Given the higher than expected rate of intention to contain and containment behaviour, it was not possible to assess and compare the impact of the different information materials as initially intended. However, we were able to identify a number of important factors that impacted on the capability, opportunity and motivation of those participants who were not keeping their cat fully contained and link them to appropriate behaviour change strategies. Although it is important to provide cat adopters with advice about how to contain their cats properly, these results also highlighted the importance of focusing attention on other behaviour change strategies that address the particular barriers to those cat-owners who were unsuccessful in keeping their cat contained at all times.

## Figures and Tables

**Table 1:** The 20 potential behavioural factors influencing cat owners’ containment behaviour identified from the literature that were used in the questionnaire.

Type	Behavioural Factor
Capability	Cat howls too much if unable to get outside
	Cat uses existing doggy door
	People leave door open and let cat out
Opportunity	Kitty litter too expensive
	Home is too small
	Too expensive to build an enclosure
	Neighbours do not want cat-proof fence
Motivation	Believe containment prevents predation
	Believe containment prevents fighting with other cats
	Believe containment prevents traffic injury
	Believe containment prevents stealing
	Believe containment reduces being lost
	Believe containment prevents injury by cat hater
	Believe containment prevents disease transmission
	Do not like urine smell in house
	Believe cat needs to roam
	Believe cat will get fat if contained
	Believe their cat does not like being contained
	Believe cat will be destructive if contained
	Believe not all cats kill wildlife

**Table 2:** Rating means and ranges of the content of the provided booklet by those participants who were keeping their cat fully contained (N = 26), contained at night only (N = 3) and not contained (N = 2). Rating scale: 5 = highly agree, 4 = agree, 3 = somewhat agree, 2 = slightly agree, 1 = do not agree.

Measures	Fully Contained (N = 26) Mean (Range)	Contained at Night (N = 3) Mean (Range)	Not Contained (N = 2) Mean (Range)
Easy to read	4.3 (3–5)	4 (4)	4.5 (4–5)
Credible	4.3 (3–5)	4 (4)	4.5 (4–5)
Clear steps	4.3 (3–5)	3.7 (3–4)	4.5 (4–5)
Encouraging	4.3 (3–5)	3.7 (3–4)	4 (4)

**Table 3:** Summary of demographic variables across the three containment profiles.

Variable	Contained Fully (N = 63)		Contained at Night (N = 5)		Not Contained (N = 4)		Group Differences
	**Mean**	**SD**	**Mean**	**SD**	**Mean**	**SD**	**F**
Age (years)	37.0	13.2	32.0	12.4	41.0	3.6	0.56
	**n**	**Z_Resid_**	**n**	**Z_Resid_**	**n**	**Z_Resid_**	**Χ^2^**
Gender:							1.65
Male	14	−0.7	1	−0.2	2	1.3	
Female	49	0.7	4	0.2	2	−1.3	
Household:							2.42
Adults only	37	1.5	2	−0.8	1	−1.3	
Family (children)	25	−1.5	3	0.8	3	1.3	
Home ownership:							4.22
Own	35	−1.9	5	1.9	3	0.6	
Rent	28	1.9	0	−1.9	1	−0.6	
Locality:							1.79
Inner city	16	0.9	1	−0.2	0	−1.1	
Suburb	44	−1.1	4	0.3	0	1.2	
Rural	3	0.5	0	−0.4	4	−0.3	
Building type:							6.17
Flat	17	1.8	0	−1.3	0	−1.1	
Duplex	10	1.3	0	−0.9	0	−0.8	
House	36	−2.5	5	1.8	4	1.6	
Outdoor space:							13.87
None	3	0.7	0	−0.5	0	−0.4	
Balcony	13	1.5	0	−1.1	0	−1.0	
Courtyard	12	0.4	0	−0.7	1	0.3	
Backyard	32	−1.6	5	1.8	3	2.7	
Acreage	3	0.7	0	−0.5	0	−0.4	
Other pets:							12.02 * (r =0.30)
Dogs	20	0.6	2	0.5	0	−1.4	
Other	3	−2.9	1	1.0	2	3.1	
None	40	1.1	2	−1.0	2	−0.5	

* Significant at *p* < 0.05, r—Pearson’s correlation coefficient; r = 0.30 indicates effect size is medium [28], Z_Resid_, Adjusted standardized residual, where Z^Resid^ > |2| is significant at *p* < 0.05.

## Supplementary Materials

The following are available online at https://www.mdpi.com/2076-2615/10/7/1214/s1, S1: PDF of the RSPCA booklet ‘Keeping your cat safe and happy at home’. S2: Surveys used in the study.
