# Supplementary material for: Understanding the Relationship between Intention and Cat Containment Behaviour: A Case Study of Kitten and Cat Adopters from RSPCA Queensland
Source: Animals (Basel). 2020 Jul 16;10(7):1214. doi: 10.3390/ani10071214 (PMC7401661; doi:10.3390/ani10071214)
Supplement: Supplementary file 1 [file animals-10-01214-s001.zip › animals-848291-supplementary S1.pdf]

keeping your cat SAFE AND happy AT HOME

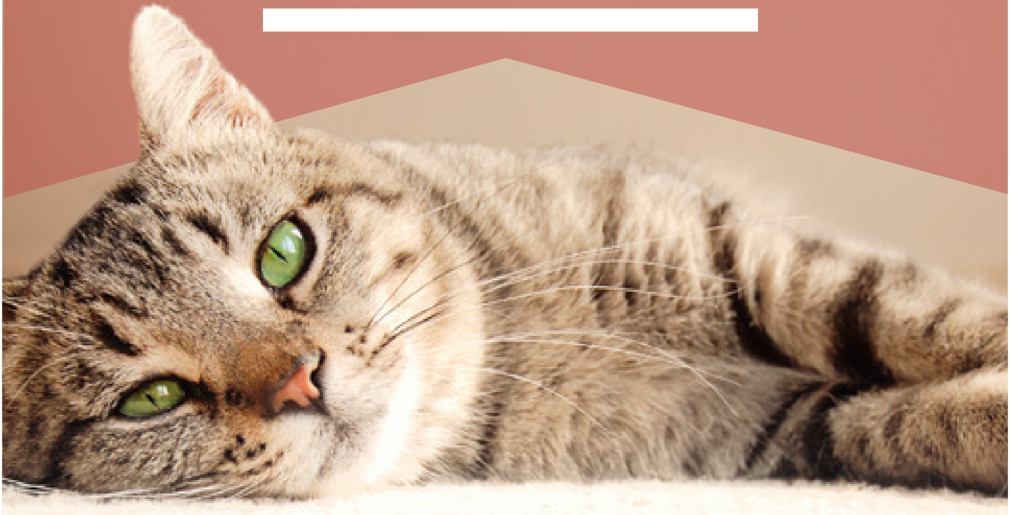

RSPCA

for all creatures **great** & **small**

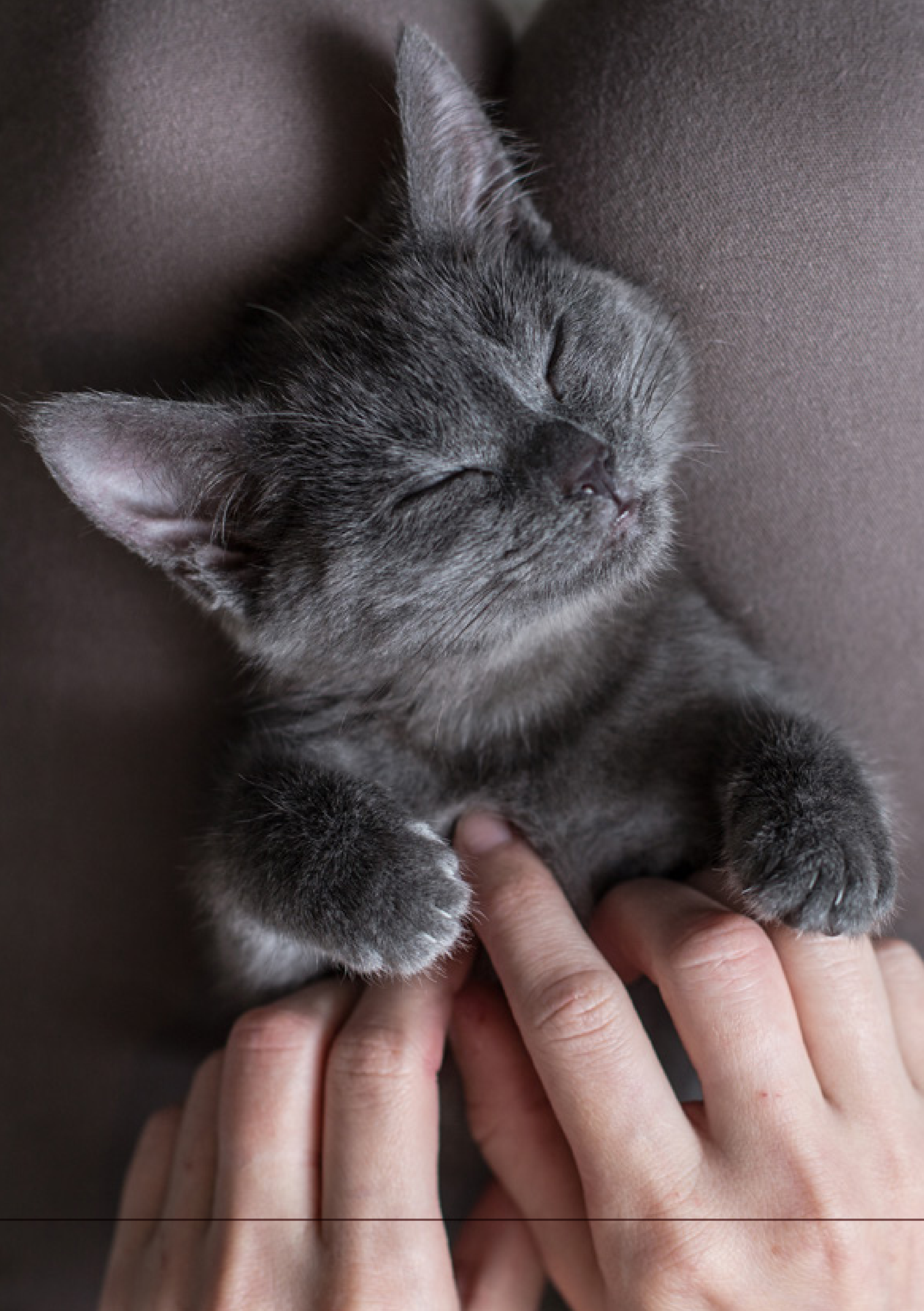

Introduction

Thank you for providing a loving home for your new feline friend!

As you both settle in and get to know each other, now is a good time to consider an important step in giving your cat a safe and happy home life. The idea that all cats need to roam outdoors has changed, with many owners now providing a safe and suitable environment for their cat at home all day, every day. Keeping your cat contained and content at home means providing for all their needs, including aspects that would previously have been met by being outdoors. Meeting these needs in other ways requires forward thinking and a little imagination and commitment. But you needn't go it alone: we're here to help explain all about containing cats to help you and your cat live at home together in harmony.

PART 1

SAFE AT HOME, DON'T LET ME ROAM

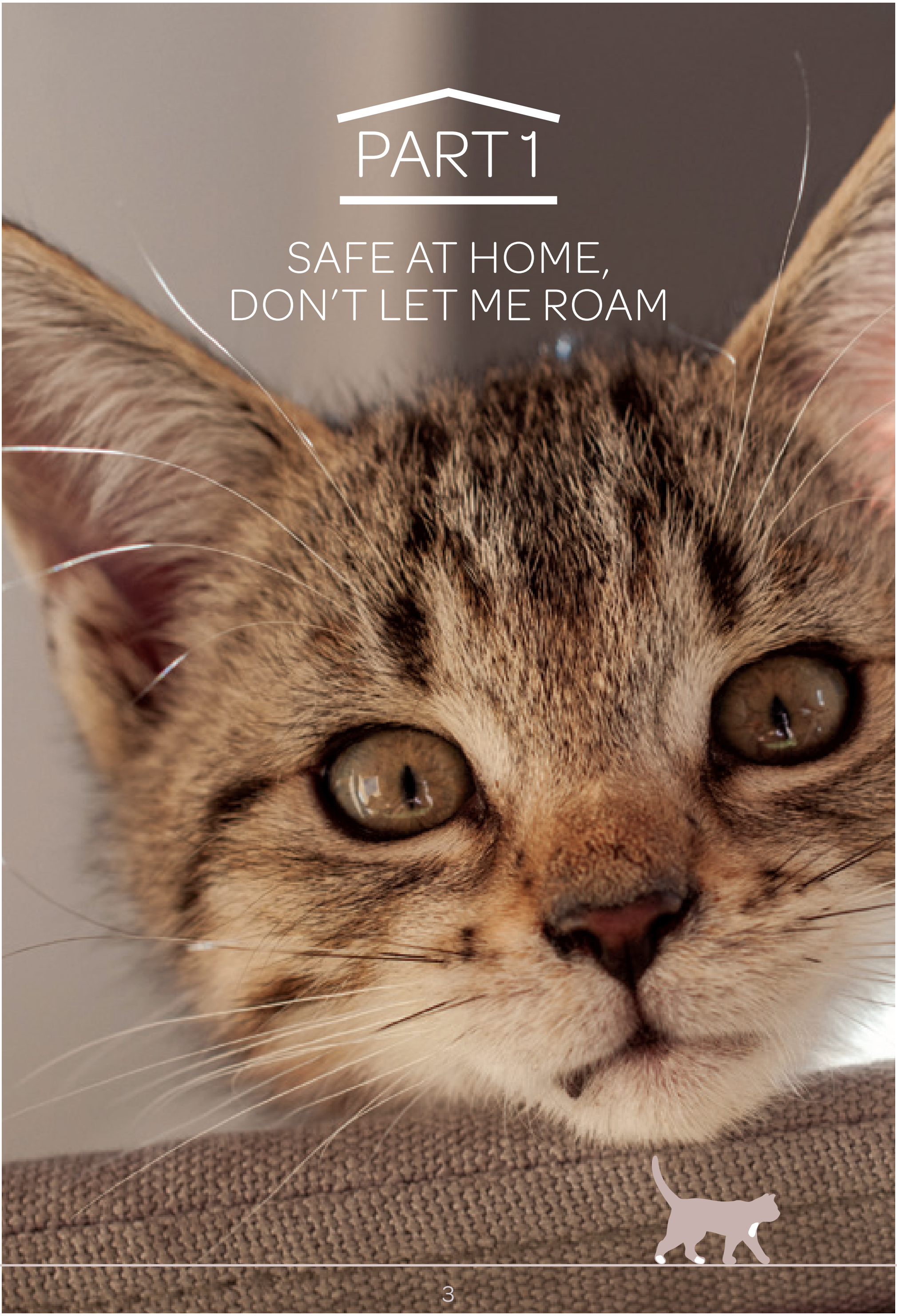

What is cat containment?

Containing your cat means completely preventing them roaming from your property at any time, day or night. This can be achieved by keeping your cat indoors, or a combination of indoors and an outdoor enclosure; or cat-proof fencing around an outdoor area. In other words, keeping your cat safe at home and not letting them roam.

Why should I keep my cat contained?

By keeping your cat safe at home you will be:

- reducing the risk of them getting sick, being hurt or dying in an accident
- enjoying more quality time together
- preventing them from harming or killing other animals
- avoiding problems with your neighbours
- giving them a better chance to enjoy a longer, healthier life.

THE RISKS OF ROAMING FREE

Keeping your cat at home avoids the risk of disease, injury or death from:

- being hit or run over by a car
- being attacked by a dog
- fighting with other neighbourhood cats
- acquiring a serious infection, such as Feline Immunodeficiency Virus (FIV) or Feline Leukaemia Virus (FeLV), which can be fatal or have life-long consequences
- eating poison baits intended for other animals such as rats or foxes, or eating rats or mice that have ingested poison baits. Sadly, pet cats may also be victims of intentional poisoning by people that consider them to be pests
- being stolen or abused
- picking up ticks or fleas and acquiring related illnesses such as tick paralysis
- being bitten by a venomous snake.

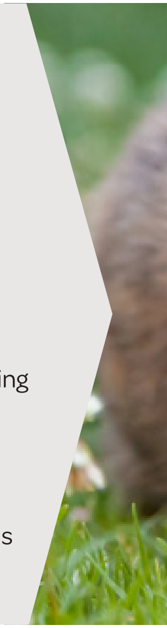

Will my cat be happy staying at home?

Your newly adopted cat is more likely to settle in to an indoor lifestyle if you keep them at home from the beginning. They will find it harder if you let them outside and then later try to contain them. Kittens generally adapt well to an indoor lifestyle if they have been contained from an early age. Newly acquired cats should always be contained during the settling-in period anyway, as they may run-off or become lost if allowed outside.

If you have an existing cat who roams then try to introduce them to being contained gradually (e.g. initially keep them inside at night, then gradually increase the time they are confined during the day). It is essential to provide lots of distractions like new hiding and resting areas in the house, a variety of toys, and extended play sessions every day.

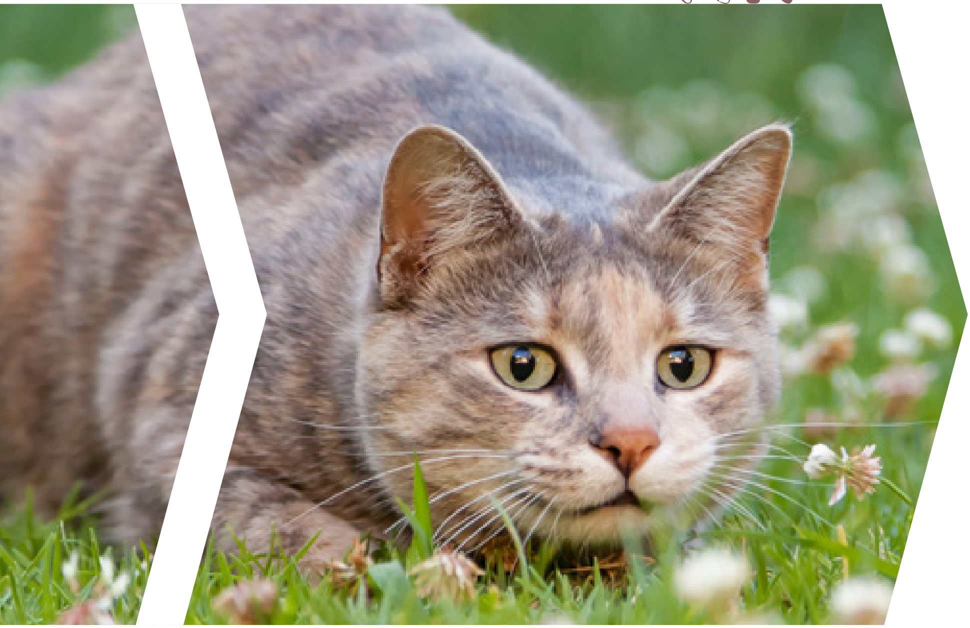

How do I keep my cat at home?

There are three main options for keeping your cat contained:

1. Totally indoors

- This is often the easiest and cheapest option, and can work well as long as you are able to provide for all your cat's needs within your home. That means providing enough space and different areas for toileting, sleeping, hiding, scratching and playing as well as access to food and water.
- To help your indoor cat benefit from exposure to outdoor sights, smells and sounds, you can install cat-proof fly screens so that windows and doors can be opened without your cat escaping.

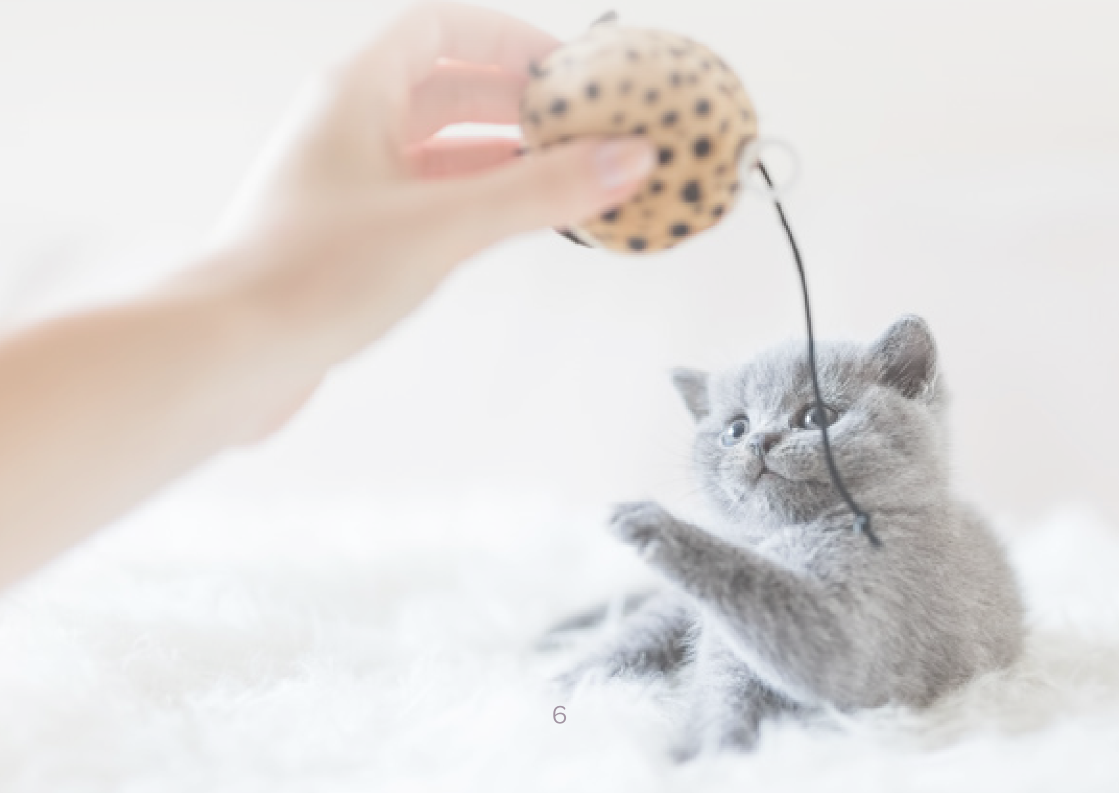

2. Indoors with an outdoor enclosure

- Providing a secure outdoor area is a great way of expanding your cat's environment. This can be attached to your cat's indoor space via a window or cat flap, or you can build a free-standing enclosure. Make sure the enclosure is both escape-proof and snake-proof.
- Having an outdoor run linked to the indoors means your cat can choose where they spend their time and there is less risk of accidental escape than if you are moving them into a separate enclosure. If you have a balcony or veranda which can be made escape-proof, this is a great option for you.
- If you build a separate outdoor enclosure, remember your cat will need the same essential resources they have indoors, otherwise they may feel frustrated at being outside but not free to roam. Make sure you include a variety of platforms at different heights, hiding places and scratching posts, food, water and more than one litter tray.
- It's best not to place the enclosure where dogs or other cats can be a threat. That includes places where your cat can see other cats or dogs (e.g. over the next door fence) even if the other animal cannot actually get to your cat.

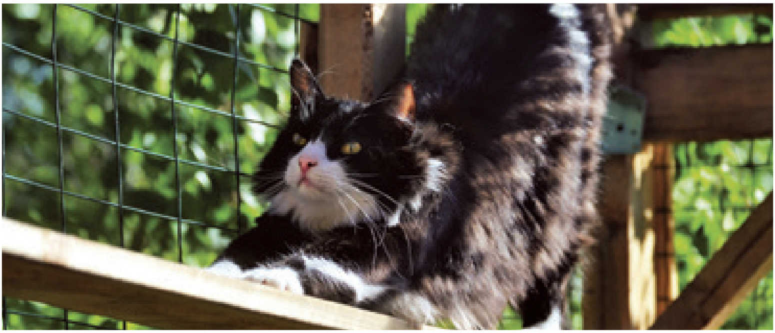

In an outdoor enclosure, make sure you include a variety of platforms at different heights, hiding places and scratching posts, food, water and more than one litter tray.

3. Indoors with an outdoor space surrounded by an escape-proof fence

- With this option your cat can truly get the best of indoor and outdoor living while still being safe. There are plenty of ideas and products available to help you make your yard or garden escape-proof.
- If you have an existing continuous solid fence around your backyard, you will need to modify or extend the fence to make it escape-proof. Alternatively you can erect a new solid fence or use netting to create a secure space.

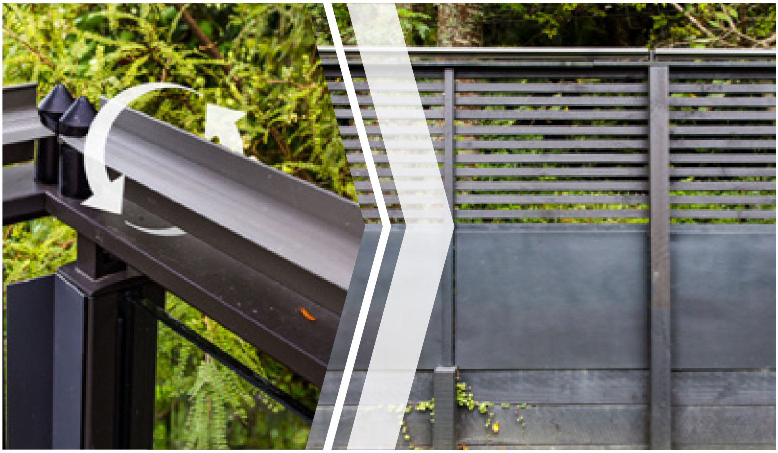

LEFT: A cat-proof rolling bar attached to the an existing fence prevents cats from climbing over.

RIGHT: An existing fence, increased in height to more than two metres with a rolling attachment.

CAT-PROOFING A BACKYARD

- To prevent your cat escaping over a continuous solid wooden or metal fence, it must be modified at the top in all places to prevent your cat gaining purchase and climbing over. You can achieve this using smooth metal or plastic sheeting, rolling cylinders or inward-inclined wire.
- Don’t forget to check for anything that might lead to an escape such as gaps in the fence, around gates or where the fence meets the building, overhanging trees and tree trunks near the property boundary.
- If you have any trees close to the fence, a simple way to stop climbing is to fix a smooth metal or plastic band around the trunk at least two metres off the ground.

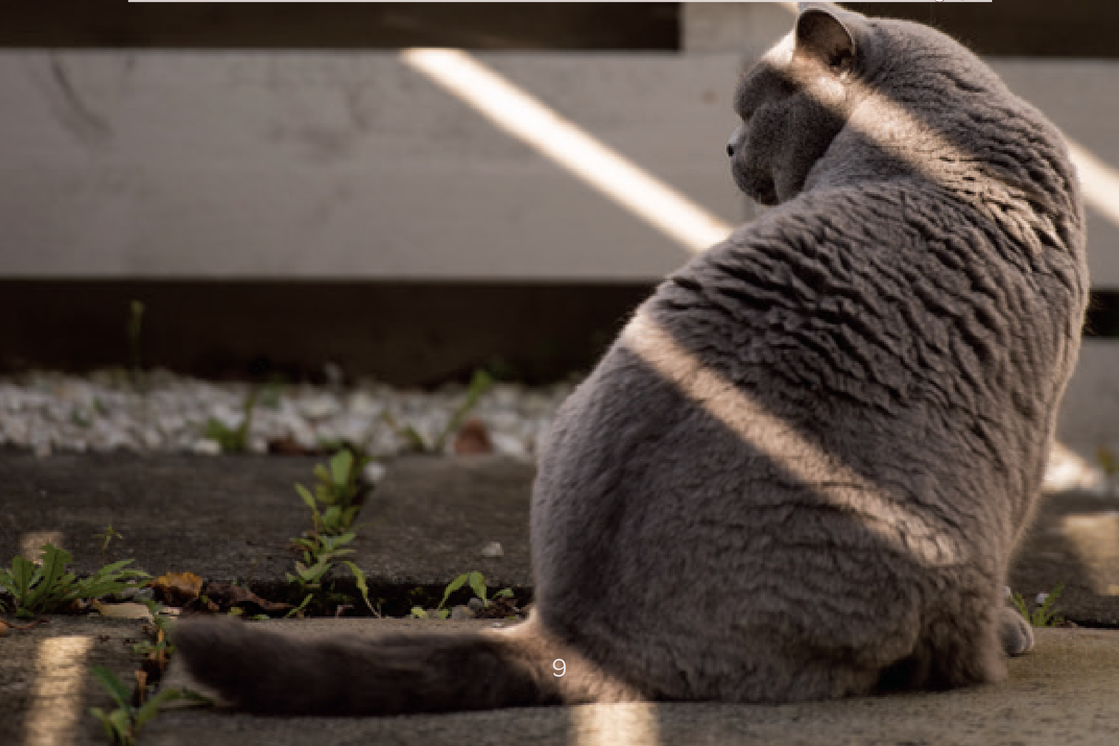

CAT-PROOFING A BALCONY OR VERANDAH

- Rigid netting can be used if the top of the balcony is cat-proofed or fully enclosed. Otherwise the netting should be floppy (but not stretchy) to discourage climbing.
- To avoid your cat getting their paws, claws or even their head caught in the net, the openings should be about 50mm wide.
- If a net extension is being added to an existing fence, the total height needs to be at least two metres or your cat may jump over it.
- Keep the net in good condition by reinforcing it along attachment points and making sure it can't be chewed.

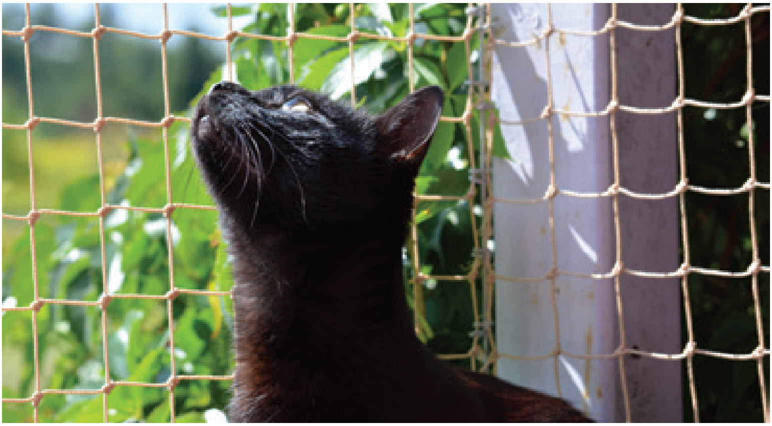

Cat-proofing a balcony or verandah allows access to fresh air, sunshine and all the sights and smells that interest your cat.

PART 2

KEEPING ME HAPPY AND HEALTHY AT HOME

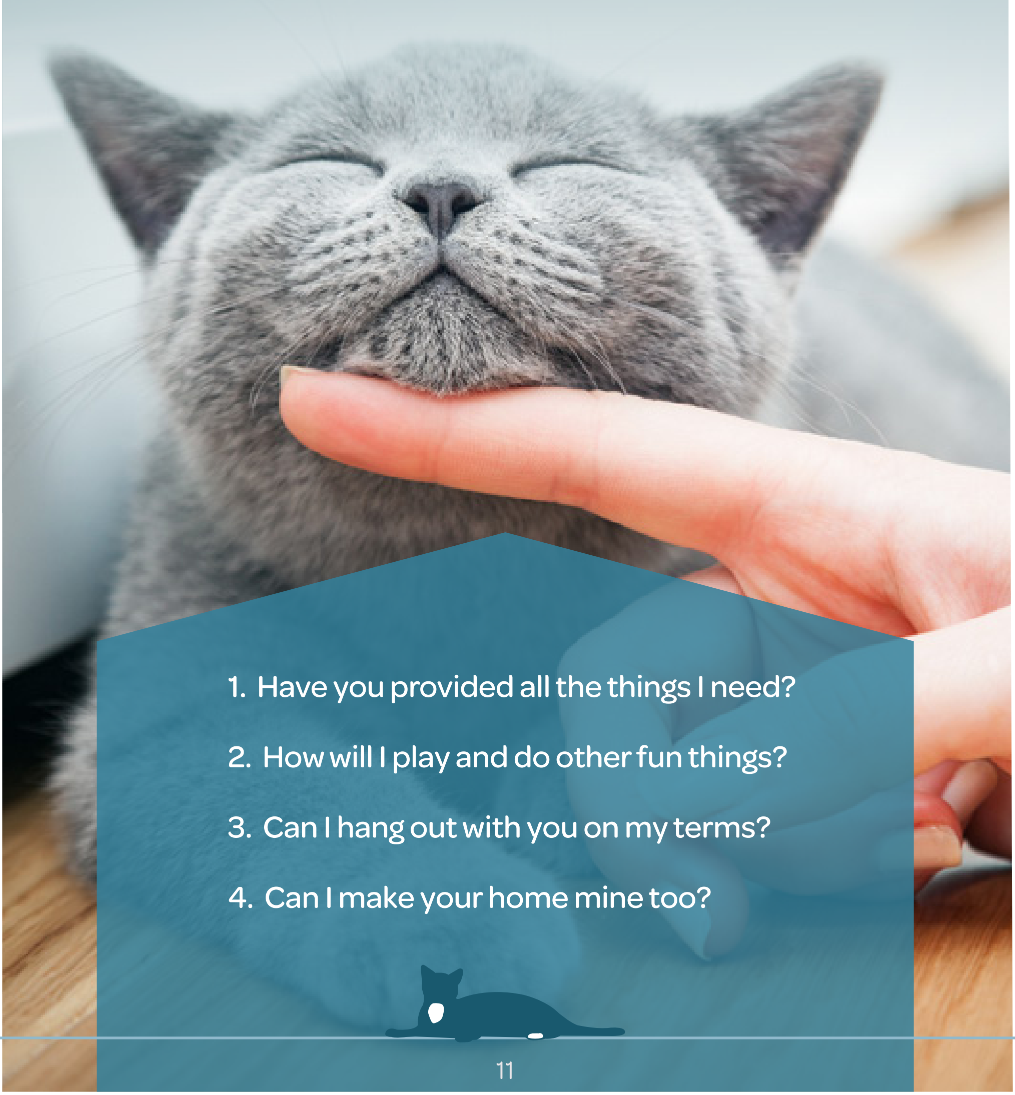

1. Have you provided all the things I need?
2. How will I play and do other fun things?
3. Can I hang out with you on my terms?
4. Can I make your home mine too?

While there are many benefits of keeping your cat at home, it also means that you have complete responsibility for and control over their environment. Their choice about where they rest, play and toilet, and opportunities to perform normal behaviours such as hunting, climbing, scratching and exploring will be limited. So it's essential that the spaces you provide meet your cat's needs and encourage them to undertake activities that they enjoy and will promote their well-being. If you don't provide an appropriate environment, then your cat may develop health and behavioural problems. Such problems can be difficult to resolve, but fortunately they can be relatively simple to prevent.

1. Have you provided all the things I need?

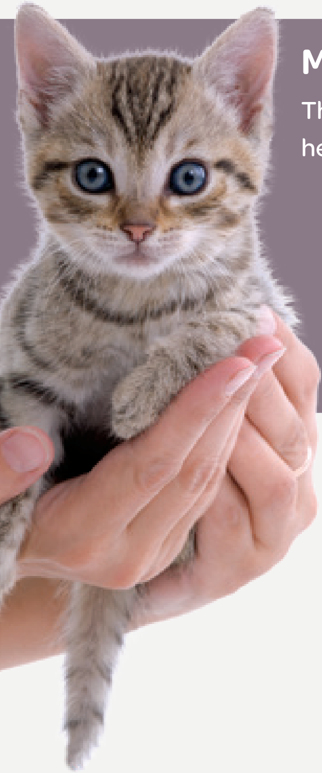

My five basic needs

The main things your cat needs to keep them happy and healthy are choice and variety in these five basic resources:

1. **resting and hiding places**
2. **food**
3. **water**
4. **toileting areas (litter trays)**
5. **places to scratch.**

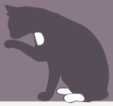

All you have to do is offer more than one choice for each resource, then let your cat decide for his or herself. As a guide, for one cat you will need two litter trays, two feed stations, two water bowls, three different types of scratching surfaces and three resting/hiding places in different locations. If you have several cats, you'll need to provide more resources in more locations as cats don't always like to share!

Resting and hiding places

Private, safe and quiet places – all cats need these to retreat to and to help them feel secure and happy. They love hiding and you will be surprised at some of the spots they choose - shelves, cupboards, a bed or box on top of a chest of drawers or wardrobe, inside a wardrobe, or underneath a bed. Offering some elevated spots to encourage jumping and climbing will help keep joints and muscles healthy. You can also buy cat furniture with different levels and surfaces and many have a built-in hiding box. Other options include: a cat carrier left open and wooden, plastic or cardboard boxes. Remember to make your cat's safe places comfortable with a soft bed – they will love you for it!

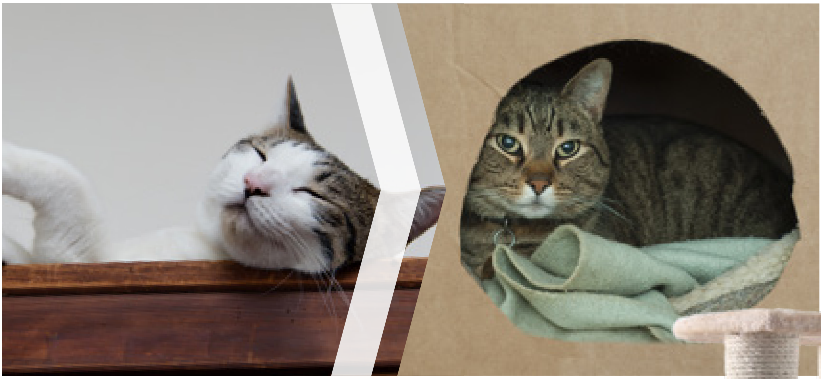

ABOVE: Existing furniture or container can offer great safe places.

RIGHT: Cat furniture is a great way to provide plenty of choices of height, location and hiding places.

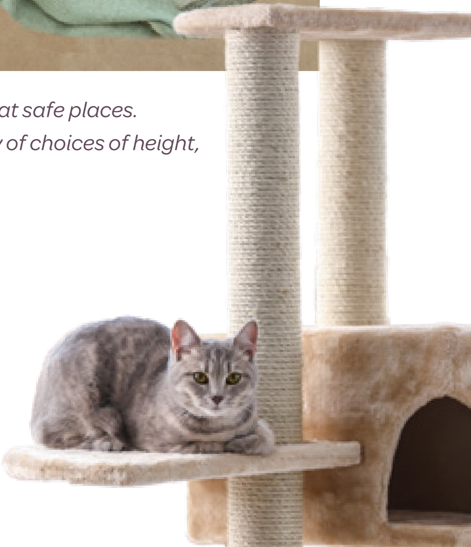

Food

It's not just what you feed your cats, but where and how you feed them, that's important. Making feed time fun by giving frequent small amounts of food, introducing different textures and flavours, and placing food in different locations, will keep your cat in good shape for body and mind. Make sure you offer food in a separate place from water bowls and toilet areas.

> **Prevent poisoning:** make sure that you don't have any toxic plants (such as lilies) in your home as these can be fatal. You can read about what plants are poisonous to cats on the RSPCA Knowledgebase.

Water

Cats often have individual preferences for what they like to drink from and where they like to drink. Many prefer ceramic or glass rather than plastic bowls. Give your cat several bowls in different spots filled with clean, fresh water and take note of what they prefer – you'll probably find that bowls from your own kitchen are the most popular!

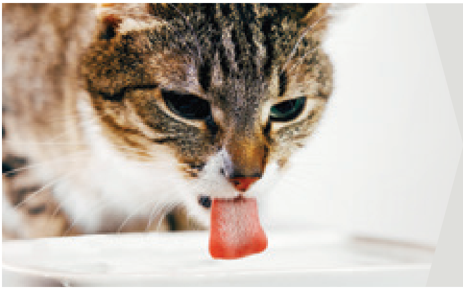

Cats prefer a wide, shallow bowl filled to the brim as this avoids their whiskers touching the sides.

Litter trays

It's more important than you might think to help your cat feel comfortable when they need to toilet. Sadly, inappropriate toileting is one of the major reasons for cat surrender. The good news is that there are a several things you can do to avoid toileting becoming a problem.

TOILETING TIPS

- Place litter trays in a quiet area away from food and water bowls, i.e. corner of a room or in a gap bordered by furniture.
- You need two trays for one cat, and another tray for each additional cat, all in different places.
- The trays need to be large enough for your cat to comfortably dig, turn around and squat – that means at least 1.5 times the length of your cat. Many commercial litter trays are too small, but it is easy to make your own using modified plastic storage containers.
- Cats need to dig, so make sure you fill the tray with litter to a depth of at least 3 cm.
- Some cats prefer a sandy or clay-based litter, others like wood pellets or crystals. You can try out different litter types and see what your cat likes to use.
- Ideally you should spot clean the litter tray every day, and replace the litter once a week. It is best not to use scented litter or to leave soiled litter for too long as this may put your cat off using their tray.

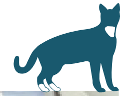

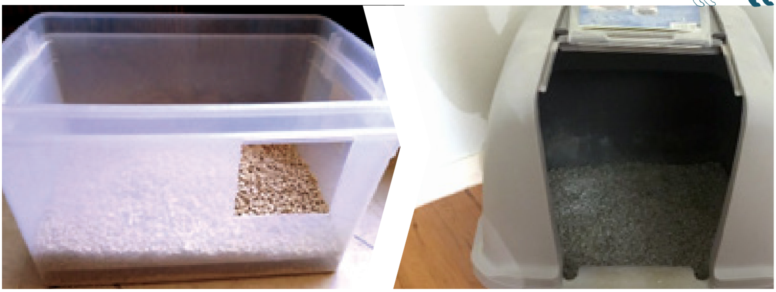

Plastic storage containers make ideal litter trays.

Places to scratch

Cats love and need to scratch – it helps spread their scent and keep their claws healthy. Providing a variety of horizontal and vertical scratching posts in different locations and with different surfaces will keep them busy and reduce the chance of them scratching other furnishings. Long vertical posts are good as they also allow your cat to enjoy a full body stretch.

2. How will I play and do other fun things?

Stalking, chasing and pouncing are what cats need and love to do and are part of being a natural hunter. Even though the great outdoors is off-limits, cats staying at home can still do all these things, and it's easy to provide them with substitutes for hunting.

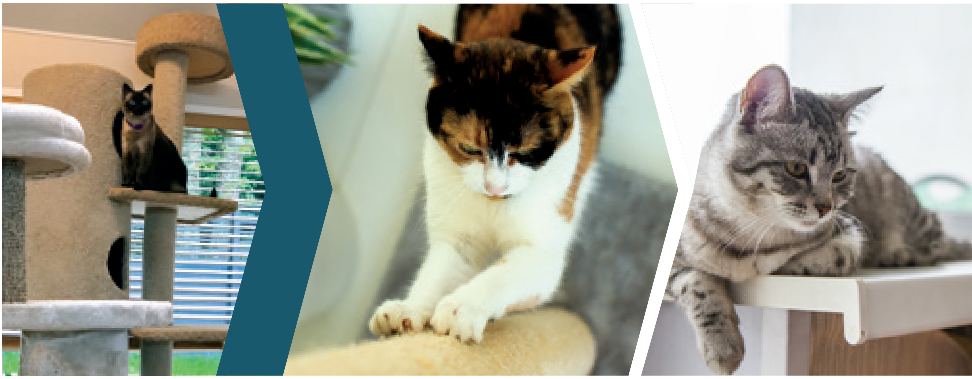

LEFT: Make sure your cat has both vertical and horizontal scratching areas.
CENTRE: A long vertical scratching post allows your cat to fully stretch.
RIGHT: Jumping onto windowsills and tables keeps your cat physically and mentally healthy.

Food games

- Hide food in different locations for your cat to search for and find.
- Throw individual pieces of dry food for your cat to chase.
- Try out a food ball or a puzzle feeder where your cat has to play to obtain the food.

Playtime

Some cats prefer to jump in the air, others prefer to run on the ground, some prefer bird-like toys and others mouse or insect-like toys. Learn your cat's preferred style of play and find the best toys and activities to match it.

- Let your cat catch the toy occasionally to give satisfaction.
- Occasionally reward a 'capture' with a treat.
- Vary play sessions, rotate between different toys, and hide toys to provide more stimulation and prevent boredom.

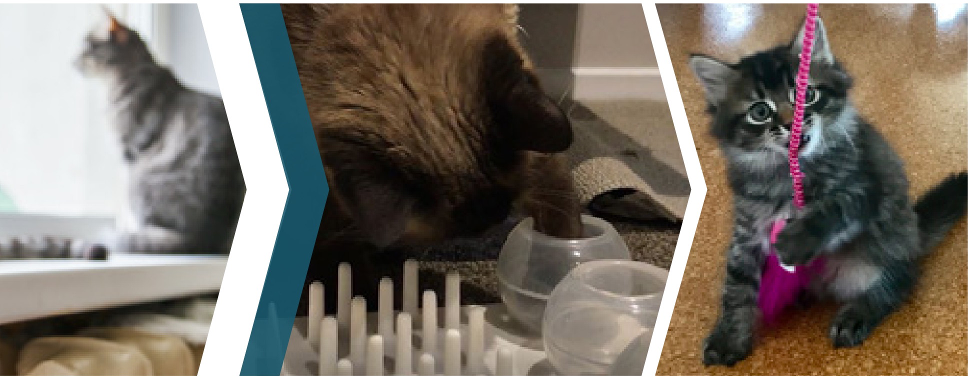

ABOVE: Food puzzles relieve boredom.
RIGHT: Toys can be moved in the air or along the ground like a bird or mouse.

3. Can I hang out with you on my terms?

We all know how independent cats are, but this is one of the things we love about them. All this means is that they want to 'call the shots' when it comes to hanging out with you or other people. You just have to follow the lead: for example, they may like to come up to you and rub against your legs, but may object if you go to pick them up. But that's okay – they will let you know what they want and when they want it. Respecting this helps build trust and harmony between you and your cat.

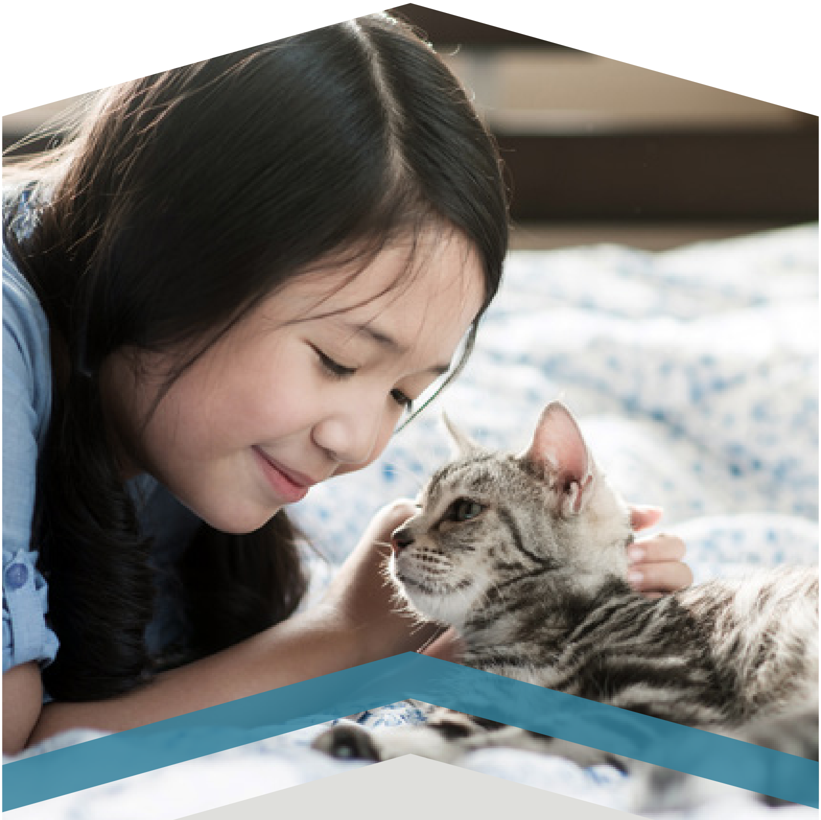

Enjoy some daily 'cat time'.
Always allow your cat to initiate and guide the type of contact.

Allow your cat to scent mark, especially new items. You should avoid cleaning 'scent mark' areas.

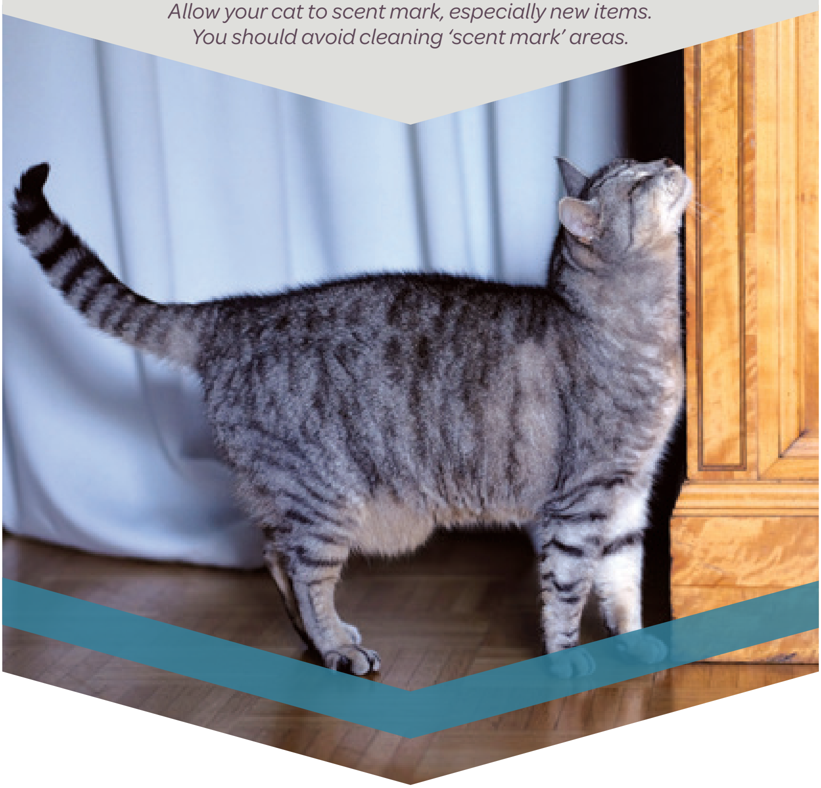

4. Can I make your home mine too?

All cats need to 'mark their territory', which means they will scent mark your home to make it theirs too. You will see them rubbing their face and body on furniture, doorways and other places. It is really important they do this as it helps them to feel safe and secure which means they will be happier and healthier. It's best to avoid cleaning these scent marks off – at least for a while.

Common behavioural problems

If you've followed this advice so far, you should be on the right track for a happy and healthy future with your cat. But things don't always go according to plan, especially when cats are entering a new home. If that's the case, you might encounter one of these common problems:

- howling and yowling
- scratching furniture
- peeing or pooing in the wrong place
- being aggressive towards other cats or humans.

These behaviours are more likely to develop if your cat has become stressed because their basic needs are not being fully met. As a first step, carefully check that you've covered all the points we've outlined here and that they have choices in where they feed, drink, rest, hide, scratch, toilet and play. Observe your cat to see how and where they spend their time and what choices they make. If there is anything that you haven't got right, then fix this straight away. If problems persist, you should seek veterinary and/or behavioural advice as soon as possible.

Living in harmony

Keeping your cat happy and healthy without roaming from home, will help ensure harmony for everyone – you, your cat, neighbours and local wildlife.

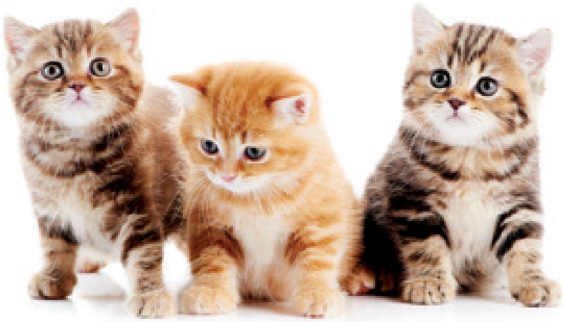

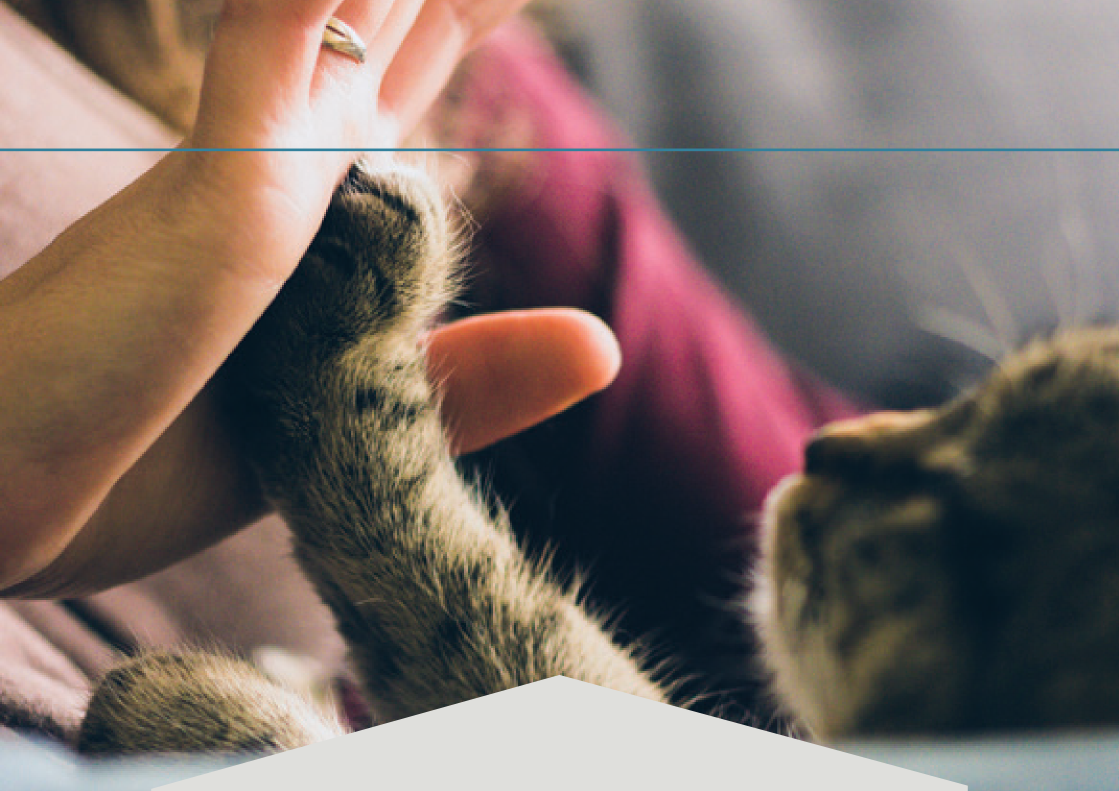

Useful resources

- RSPCA Knowledgebase: kb.rspca.org.au
- Safe cat, safe wildlife: safecat.org.au
- Indoor Pet Initiative: indoorpet.osu.edu
- International Cat Care: icatcare.org
- American Association of Feline Practitioners resources:
  - catfriendly.com
  - catvets.com/guidelines/client-brochures
- Food puzzles for cats: foodpuzzlesforcats.com
- RSPCA World for Pets online shop: worldforpets.com.au

This project has received support from the Elizabeth Mary William Trust, managed by Equity Trustees.

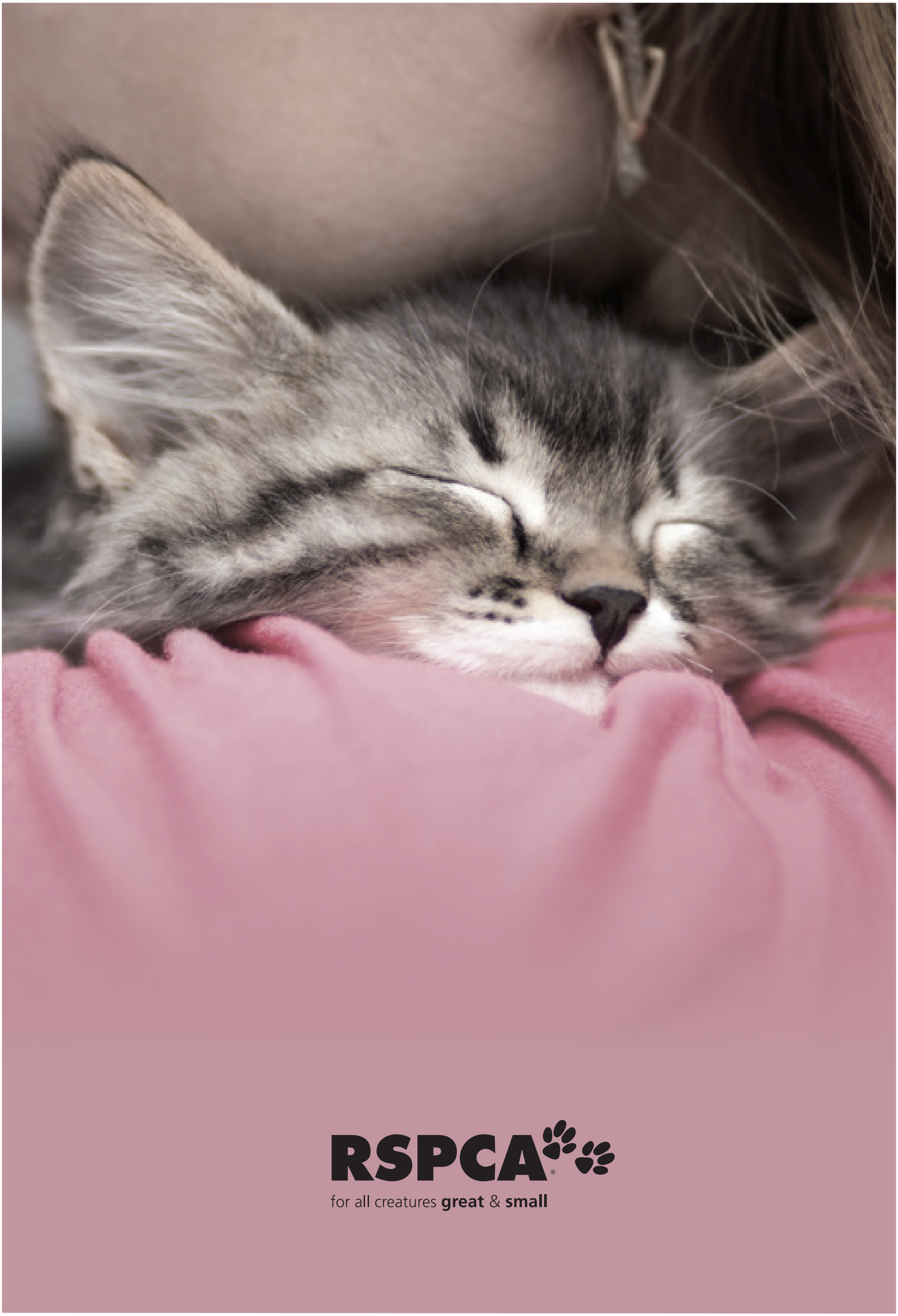
RSPCA
for all creatures great & small
