# Supplementary material for: Understanding the Relationship between Intention and Cat Containment Behaviour: A Case Study of Kitten and Cat Adopters from RSPCA Queensland
Source: Animals (Basel). 2020 Jul 16;10(7):1214. doi: 10.3390/ani10071214 (PMC7401661; doi:10.3390/ani10071214)
Supplement: Supplementary file 1 [file animals-10-01214-s001.zip › animals-848291-supplementary S2.pdf]

Supplementary Materials 2: Surveys used in the study

Survey for Participants

Keeping cats safe at home

Survey 1

AID: ______________________________

Adopter's first name: ____________________________________ Date: _____________________

Q1a: Do you currently have any other cats in your household?

☐ **No** (skip to Q2) ☐ **Yes,** Number of cats _____________________

Q1b: Please select the response that best describes your current cat's normal living arrangements

☐ Lives outside all the time

☐ Allowed inside or outside at any time

☐ Kept indoors at night, but allowed outside during the day

☐ Kept indoors most of the day but allowed outside for short periods

☐ Kept indoors all the time

Q1c: If your cat/s go outside, do they have

☐ Unrestricted access to outdoors

☐ Restricted access to outdoors e.g. kept in a cat run / enclosure /courtyard from which they can't escape, supervised all the time, or walked on a lead.

☐ Not allowed outside

☐ Other, please describe ____

Q1d: Will your new cat/kitten have the same living arrangements as you answered above in Qs 1b and 1c (after the settling-in period is over)?

☐ **No** (go to Q2a) ☐ **Yes** (end survey)

Keeping cats safe at home

Survey 1

Q2a: Which response best describes the living arrangements for your new cat / kitten (after the settling-in period is over)

- ☐ Lives outside all the time
- ☐ Allowed inside or outside at any time
- ☐ Kept indoors at night, but allowed outside during the day
- ☐ Kept indoors most of the day but allowed outside for short periods
- ☐ Kept indoors all the time

Q2b: If your new cat is allowed outside, will they have

- ☐ Unrestricted access to outdoors
- ☐ Restricted access to outdoors e.g. kept in a cat run / enclosure /courtyard from which they can't escape, supervised all the time, or walked on a lead.
- ☐ Not allowed outside
- ☐ Other, please describe ______________________________

OFFICE USE ONLY

Research Group (Adoption counsellor to tick appropriate box)

Control: ☐ Usual counselling session conducted

Treatment: ☐ Additional information and encouragement on cat containment

Online survey questions

What is your age?

What gender do you identify as?

- O Male (1)
- O Female (2)
- O Other (3)

What is your postcode?

How many people live in your household?

Adults>18 years (1)	
Children 12-18 years (2)	
Children (3)	

Do you own or rent your home?

- O Own (1)
- O Rent (2)
- O Other (please specify) (3) ____

Do you have other types of pets at home (other than cats)? (please select all appropriate responses)

- ▢ Dogs (1)
- ▢ Birds (2)
- ▢ Rabbits (3)
- ▢ Guinea pigs/rats/mice (4)
- ▢ Reptiles (5)
- ▢ Fish (6)
- ▢ Other (please specify) (7) ____________________

Which <u>ONE</u> of the following best describes your current locality?

- O Inner city (1)
- O Suburban (2)
- O Residential in rural town (3)
- O Peri-urban/semi-rural (4)
- O Rural property (5)
- O Other (please specify) (6) ____________________

How would you describe your dwelling?

- O Apartment/Unit (1)
- O Townhouse (2)
- O Duplex/Triplex (3)
- O House/Stand-alone dwelling (4)
- O Other (please specify) (5) ____________________

What type of outdoor area does your home have? (you may choose more than one option)

- ☐ I do not have an outdoor area (1)
- ☐ A balcony with cat access to ground level (2)
- ☐ A balcony with no cat access to ground level (3)
- ☐ An enclosed (fenced) courtyard (4)
- ☐ Courtyard with no fence (5)
- ☐ An average-size suburban backyard—fenced (6)
- ☐ An average-size suburban backyard with no fence (7)
- ☐ Acreage (8)
- ☐ Other (please specify) (9) ____________________

Please select the response that best describes your newly adopted cat's normal living arrangements.

- O Lives outside all the time (1)
- O Allowed inside or outside anytime (2)
- O Kept indoors at night but allowed outside during the day (3)
- O Kept indoors most of the time, but let outside for short periods (4)
- O Kept indoors all the time (5)

What do you imagine when people talk about cat containment? (choose all responses that apply)

- ▢ Keeping the cats indoors all the time (1)
- ▢ Keeping the cats indoors only at night (2)
- ▢ Allowing the cat outdoor access for only short periods (3)
- ▢ Allowing the cat outdoor access in a yard with a solid timber or metal fence (4)
- ▢ Allowing the cat outdoor access only in a cat enclosure (cat run) or specially constructed cat-escape proof yard (5)

Please read the following definitions before completing the remaining questions. Cat containment: when a cat is prevented from leaving their home property at any time (day and night) by being contained within the house and/or outdoors through a cat enclosure, a cat escape-proof fence, balcony netting or other safe, humane and effective method. **Cat enclosure:** an enclosed area (such as a purpose-built run or netted balcony area) where your cat(s) can access the outdoors but cannot roam. **Cat escape-proof fence:** a specially constructed fence that cats cannot jump or climb over and prevents your cat(s) from leaving your backyard or property. Standard backyard fences are NOT cat-proof.

What do you see as the benefits of keeping your cat on your property all the time?

If you are not keeping your cat contained to your property all the time (day and night), what are your reasons?

Please rate how much you agree with the following reasons why cats should be contained.

	Do not agree (1)	Slightly agree (2)	Somewhat agree (3)	Agree (4)	Strongly agree (5)
To prevent predation on wildlife (1)	O	O	O	O	O
To prevent fighting (2)	O	O	O	O	O
To prevent injury from traffic accidents (3)	O	O	O	O	O
To prevent being stolen (4)	O	O	O	O	O
To prevent being lost (5)	O	O	O	O	O
To prevent being injured by cat haters (6)	O	O	O	O	O
To prevent getting a disease (7)	O	O	O	O	O

Please rate how much you agree with the following reasons why cats cannot be contained.	Do not agree	Slightly agree (2)	Somewhat agree (3)	Agree (4)	Strongly agree (5)	Not applicable (6)
Cats don't like it (1)	O	O	O	O	O	O
Home is too small (2)	O	O	O	O	O	O
Cats need to roam and/or hunt (3)	O	O	O	O	O	O
Cats get fat if they don't roam (4)	O	O	O	O	O	O
Cats destroy furniture if they don't roam (5)	O	O	O	O	O	O
Don't like the smell of urine or cat poo in the house (6)	O	O	O	O	O	O
Too expensive to buy kitty litter (7)	O	O	O	O	O	O
Cats howl if they can't get out (8)	O	O	O	O	O	O
Not all cats kill wildlife (9)	O	O	O	O	O	O
Too expensive to build a cat enclosure (10)	O	O	O	O	O	O
Neighbours don't want a cat-proof fence (11)	O	O	O	O	O	O
Live in a rental and cat must be outside (12)	O	O	O	O	O	O
Live in a rental and can't build enclosure or fence (13)	O	O	O	O	O	O

Have a doggy door which cat uses (14)	O	O	O	O	O	O
Kids/visitors leave the door open and let the cat out (15)	O	O	O	O	O	O

What methods are you using to contain your cat (please select all applicable responses)?

O Kept **indoors** (1)

O A **cat enclosure**—*an enclosed area (such a purpose built run or netted balcony area) where your cat can access the outdoors but cannot roam* (2)

O A **cat escape-proof fence**—*a specially constructed fence that cats cannot jump or climb over, and prevents them from leaving your backyard or property. Normal back-yard fences are NOT cat escape-proof* (3)

Have you experienced any problems associated with containing your cat?

Please select the response that best describes your newly adopted cat's outdoor access.

When my new cat goes outside they have....

O Unrestricted access, allowed to roam freely (1)

O Restricted access, roaming is controlled (i.e., in a cat run/enclosure/courtyard from which they can't escape/supervised all the time or walked on a lead) (2)

O Other (please specify) (3) ____

Do you intend to install/use a cat enclosure in the future (i.e an enclosed area such as a purpose-built cat run or netted balcony area) where your cat(s) can access the outdoors but cannot roam?

O Definitely not (1)

O Probably not (2)

O Might or might not (3)

O Probably yes (4)

O Definitely yes (5)

Do you intend to install/use a cat escape-proof yard in the future (i.e., a specially constructed fence that cats cannot jump or climb over)?

O Definitely not (1)

O Probably not (2)

O Might or might not (3)

O Probably yes (4)

O Definitely yes (5)

On the day you adopted your cat you were sent an EMAIL from RSPCA Qld containing links to a range of cat care information, including about keeping your cat contained.
Please indicate the extent you made use of the links provided

- O Did not receive the email (1)
- O Do not recall the email (2)
- O Received the email but did not click on the links (3)
- O Did not read the information sheet relating to cat containment (4)
- O Read some of the information relating to cat containment (5)
- O Read only the sections in the cat containment information sheet that were of interest (6)
- O Read all the information in the cat containment information sheet (7)

On the day you adopted your cat, were you provided with a booklet containing information about keeping your cat contained?

O Yes (1) O No (2) O Unsure (3)

Please indicate the extent you made use of the information in the booklet provided.

O Do not recall seeing the booklet (1)

O Did not read the information (2)

O Read some of the information (3)

O Read only those sections that were of interest (4)

O Read all the information (5)

Display This Question:

If Please indicate the extent you made use of the information in the booklet provided. = Read some of the information Or Please indicate the extent you made use of the information in the booklet provided. = Read only those sections that were of interest Or Please indicate the extent you made use of the information in the booklet provided. = Read all the information

These following questions relates to the information that was provided. Please rate your agreement to each statement.

	Do not agree (1)	Slightly agree (2)	Somewhat agree (3)	Agree (4)	Strongly agree (5)
The information was easy to understand (1)	O	O	O	O	O
The information was credible (2)	O	O	O	O	O
The information provided clear steps I could follow (3)	O	O	O	O	O
The information encouraged me to take steps to contain my cat (5)	O	O	O	O	O

Have you heard about the *Safe Cats, Safe Wildlife* campaign?

O Yes (1)

O No (2)

O Unsure (3)

Display This Question:

If Have you heard about the Safe Cats, Safe Wildlife campaign? = Yes

Have you been to the *Safe Cats, Safe Wildlife* campaign website (www.safecat.org.au)?

- O Yes (1)
- O No (2)
- O Unsure (3)

Display This Question:

If Have you been to the Safe Cats, Safe Wildlife campaign website (www.safecat.org.au)? = Yes

Have you signed up to the *Safe Cats, Safe Wildlife* campaign?

- O Yes (1)
- O No (2)
- O Unsure (3)

Please use this space to add any other comments about keeping your cat happy, safe and healthy at home.

Once you are happy with your responses please press the next button so your survey results will be recorded.
